# Genetic correlates of longevity and selected age-related phenotypes: a genome-wide association study in the Framingham Study

**DOI:** 10.1186/1471-2350-8-S1-S13

**Published:** 2007-09-19

**Authors:** Kathryn L Lunetta, Ralph B D'Agostino, David Karasik, Emelia J Benjamin, Chao-Yu Guo, Raju Govindaraju, Douglas P Kiel, Margaret Kelly-Hayes, Joseph M Massaro, Michael J Pencina, Sudha Seshadri, Joanne M Murabito

**Affiliations:** 1 The National Heart Lung and Blood Institute's Framingham Heart Study, Framingham, MA, USA; 2 Section of General Internal Medicine and the Departments of Neurology, Cardiology, and Preventive Medicine, Boston University School of Medicine, Boston, MA, USA; 3 Department of Biostatistics, Boston University School of Public Health, Boston, MA, USA; 4 Hebrew Senior Life Institute for Aging Research and Harvard Medical School, Boston, MA, USA; 5 Statistics and Consulting Unit, Department of Mathematics, Boston University, Boston, MA, USA

## Abstract

**Background:**

Family studies and heritability estimates provide evidence for a genetic contribution to variation in the human life span.

**Methods:**

We conducted a genome wide association study (Affymetrix 100K SNP GeneChip) for longevity-related traits in a community-based sample. We report on 5 longevity and aging traits in up to 1345 Framingham Study participants from 330 families. Multivariable-adjusted residuals were computed using appropriate models (Cox proportional hazards, logistic, or linear regression) and the residuals from these models were used to test for association with qualifying SNPs (70, 987 autosomal SNPs with genotypic call rate ≥80%, minor allele frequency ≥10%, Hardy-Weinberg test p ≥ 0.001).

**Results:**

In family-based association test (FBAT) models, 8 SNPs in two regions approximately 500 kb apart on chromosome 1 (physical positions 73,091,610 and 73, 527,652) were associated with age at death (p-value < 10^-5^). The two sets of SNPs were in high linkage disequilibrium (minimum r^2 ^= 0.58). The top 30 SNPs for generalized estimating equation (GEE) tests of association with age at death included rs10507486 (p = 0.0001) and rs4943794 (p = 0.0002), SNPs intronic to *FOXO1A*, a gene implicated in lifespan extension in animal models. FBAT models identified 7 SNPs and GEE models identified 9 SNPs associated with both age at death and morbidity-free survival at age 65 including rs2374983 near *PON1*. In the analysis of selected candidate genes, SNP associations (FBAT or GEE p-value < 0.01) were identified for age at death in or near the following genes: *FOXO1A, GAPDH, KL, LEPR, PON1, PSEN1, SOD2, and WRN*. Top ranked SNP associations in the GEE model for age at natural menopause included rs6910534 (p = 0.00003) near *FOXO3a *and rs3751591 (p = 0.00006) in *CYP19A1*. Results of all longevity phenotype-genotype associations for all autosomal SNPs are web posted at http://www.ncbi.nlm.nih.gov/projects/gap/cgi-bin/study.cgi?id=phs000007.

**Conclusion:**

Longevity and aging traits are associated with SNPs on the Affymetrix 100K GeneChip. None of the associations achieved genome-wide significance. These data generate hypotheses and serve as a resource for replication as more genes and biologic pathways are proposed as contributing to longevity and healthy aging.

## Background

Genetic factors associated with human longevity and healthy aging remain largely unknown. Heritability estimates of longevity derived from twin registries and large population-based samples suggest a significant but modest genetic contribution to the human lifespan (heritability ~15 to 30%) [1-4]. However, genetic influences on lifespan may be greater once an individual achieves age 60 years [5]. Moreover, the reported magnitude of the genetic contribution to other important aspects of aging such as healthy physical aging (wellness)[6], physical performance [7,8], cognitive function [9], and bone aging [10] are much larger. Both exceptional longevity and a healthy aging phenotype have been linked to the same region on chromosome 4 [11,12], suggesting that although longevity per se and healthy aging are different phenotypes, they may share some common genetic pathways.

A number of potential candidate genes in a variety of biological pathways have been associated with longevity in model organisms. Genes involved in the regulation of DNA repair and genes in the evolutionarily conserved insulin/insulin-like growth factor signaling pathway [13,14] are emerging as holding great promise in the future elucidation of the underlying physiology controlling lifespan. Many of these genes have human homologs and thus have potential to provide insights into human longevity [15-20]. Although numerous candidate genes have been proposed, studies in humans are limited and initial findings often fail replication [21,22]. More recently genome-wide association studies (GWAS) have become feasible and offer a more comprehensive and untargeted approach to detect genes with modest phenotypic effects that underlie common complex conditions [23].

We had the opportunity to use the Framingham Heart Study (FHS) Affymetrix 100K SNP genotyping resource for a GWAS of longevity and aging-related phenotypes. The FHS offers the unique advantage of a longitudinal family-based community sample with participants who have been well-characterized throughout adulthood with respect to prospectively ascertained risk factors and diseases and continuously followed until death. We report several strategies for 100K SNP associations: 1) a simple low p-value SNP ranking strategy; 2) SNP selection due to associations with more than one related phenotype; and 3) SNP associations within candidate genes and regions previously reported to be associated with longevity in model organisms or humans.

## Methods

### Study sample

The genotyped study sample is comprised of 1345 Original cohort (n = 258) and Offspring (n = 1087) participants who are members of the 330 largest FHS families. The Overview [24] provides further details of this sample. With respect to aging and longevity traits, 149 deaths occurred at a mean age at death of 83 years (range 46 to 99 years) and 713 participants achieved age 65 years or greater. The Boston University Medical Center Institutional Review Board approved the examination content of Original Cohort and Offspring examinations. All participants provided written informed consent at every examination including consent for genetic studies.

### Longevity and aging phenotype definitions and residual creation

#### Age at death

Both the Original Cohort and the Offspring Cohort remain under continuous surveillance and all deaths that occurred prior to January 1, 2005 were included in this study. Deaths were identified using multiple strategies including routine participant contact for research examinations or health history updates, surveillance at the local hospital, search of obituaries in the local newspaper, and if needed through use of the National Death Index. Death certificates were routinely obtained and all hospital and nursing home records prior to death and autopsy reports (if performed) were requested. In addition, if there was insufficient information to determine a cause of death, the next of kin were interviewed by a senior investigator. All records pertinent to the death were reviewed by an endpoint panel comprised of three senior investigators. The date and cause of death (classified as due to coronary heart disease, stroke, other cardiovascular disease [CVD], cancer, other causes, or unknown cause) was recorded.

Cox proportional hazards models were used to generate martingale residuals using the PHREG procedure in SAS to perform the regression analysis of survival time from age at study entry to age at death. Models were sex-specific and adjusted for 1) birth cohort and 2) birth cohort, education, current smoking status (yes/no), obesity (body mass index ≥30 kg/m^2^), hypertension (blood pressure ≥140/90 mmHg or on antihypertensive treatment), elevated cholesterol (cholesterol > 239 mg/dL), diabetes (fasting blood sugar ≥126 mg/dL, random blood sugar of ≥200 mg/dL, or use of insulin or oral hypoglycemic agents) and comorbidity defined as CVD and cancer. Birth cohort was defined as a categorical variable for all regression models with the following categories based on year of birth: birth year prior to 1900, 1900 to 1909, 1910 to 1919, 1920 to 1929, 1930 to 1939, 1940 to 1949, and 1950 and later. All covariates were measured at study entry. Residuals from Original Cohort and Offspring participants were pooled.

#### Morbidity-free survival at age 65 years

Morbidity-free survival was defined as achieving age 65 years free of CVD, dementia, and cancer. CVD events included angina pectoris, coronary insufficiency, myocardial infarction, heart failure, stroke, transient ischemic attack (TIA), intermittent claudication and coronary or CVD death. Suspected CVD events were reviewed by a panel of three investigators who adjudicated events using previously established criteria in place since study inception [25]. A separate panel of study neurologists determined the presence of stroke or TIA and a team of at least one neurologist and one neuropsychologist determined the presence of dementia. Two independent reviewers examined records for all cancers, and the vast majority of cancer cases were microscopically confirmed with pathology reports.

Logistic regression models were used to generate deviance residuals. Models were sex-specific and adjusted for 1) birth cohort and 2) birth cohort, education, current smoking status, obesity, hypertension, elevated cholesterol, and diabetes. Covariates were defined as above for age at death. All covariates were measured at the examination closest to the participant attaining age 65 years using a 5 year window around age 65 years. Residuals from Original Cohort and Offspring participants were pooled.

#### Age at natural menopause

Natural menopause occurred after a woman had ceased menstruating naturally for one year and the age at natural menopause was the self-reported age at last menstruation. Mean age at natural menopause was similar in Original Cohort and Offspring women and the distribution of naturally menopausal ages in women in the 330 FHS families was similar to that of women in all 1643 FHS families [26,27]. The mean age at natural menopause in women in the 100K sample was 50.2 years (range 38 to 57 years) in Original Cohort women and 49.1 years (range 29 to 60 years) in Offspring women.

Crude age at natural menopause and standardized residuals from multiple linear regressions in SAS [28] that adjusted age at natural menopause for covariates of interest were used as traits for analysis. Covariates were obtained at all attended examinations prior to the onset of menopause and included mean number of cigarettes smoked per day, mean body mass index, parity (0 versus 1 or more live births), and generation (Original Cohort vs. Offspring).

### Walking speed

Walking speed was measured on Original Cohort participants at examination 27 (January 2002 through December 2003, mean age of Original Cohort at exam 27: 86.7 years) and Offspring participants attending an ancillary study to examination 7 (1999 to 2004, mean age at exam: 62.0 years). Trained technicians timed participants walking at their normal pace on a four meter course twice and subsequently asked participants to repeat the course walking at a rapid pace. The mean timed fast walk among Offspring participants in the 100K genotyping sample was 2.44 seconds (standard deviation 0.89). The timed fast walk was used for analysis. Sex-specific linear regression was used to generate residuals adjusted for age and height measured at the time of the walk.

### Biologic age by osseographic scoring system

An osseographic scoring system (OSS) was applied to hand radiographs obtained on original cohort (1967 to 1969, mean age 58.7 years) and offspring participants (1992 to 1993, mean age 51.6 years) [10]. Biologic age was then defined as the standardized residual between the OSS predicted age and the actual age. Biologic age defined by this system predicted mortality [10,29], was very heritable (h^2 ^= 0.57 ± 0.06), and a genome-wide linkage analysis was performed with LOD scores >1.8 present on chromosomes 3q, 11p, 16q, and 21q [10]. Sex- and cohort-specific ranked residuals generated from linear regression of age on log-OSS adjusted for height, body mass index, menopause, and estrogen therapy, were used for analysis.

### Genotyping

Affymetrix 100K SNP GeneChip genotyping and the Marshfield STR genotyping performed by the Mammalian Genotyping Service http://research.marshfieldclinic.org/genetics are described in the Overview paper [24].

### Statistical analysis

The statistical methods for genome-wide linkage and association analyses are described in the Overview [24].

### Association

All residual traits described above as well as the additional traits listed in Table 1 were computed using Cox proportional hazards with martingale residuals for survival traits, logistic regression with deviance residuals for dichotomous traits, and linear regression with standard residuals for quantitative traits. The full set of FHS participants with the phenotype were used to create the residuals. The residuals were used to test for association between the genotyped subset of individuals and the SNPs using additive family-based association test (FBAT) and generalized estimating equations (GEE) models as described in the Overview [24]. A total of 70,987 autosomal SNPs met the criteria of genotypic call rate ≥80%, minor allele frequency ≥10%, Hardy-Weinberg test p ≥ 0.001, and ≥10 informative families for FBAT. The number of tests with an FBAT p < 0.001, p < 0.0001, and p < 0.00001 for all phenotypes was similar to what would be expected under the assumptions that the 70,987 tested SNPs were independent and there were no true associations. The GEE tests tended to give an excess of very small p-values over what would be expected under these assumptions.

**Table 1:** Aging and Longevity Phenotypes for Framingham Heart Study 100K Project

			Exam cycle(s)		
**Phenotype Subgroup** • Trait (variable name on the website*)	**Number of Traits**	N (MV**)	**Offspring / Original Cohort**		**Adjustment**

**Survival Traits: Cox regression**					

**Survival** • Age at death (1. deathageX, 2. deathageMV)	2	1345 (1166)	Cohort & Offspring pooled		Cox regression Sex-specific 1. birth cohort 2. multivariable adjusted for birth cohort, education, smoking, obesity (BMI ≥ 30), CVD risk factors, co-morbidity measured at exam 1

**Categorical traits: Logistic regression**					

• Survival past the ALE (1. deathpastALEX, 2. deathpastALEMV)	2	1345 (1166)	Cohort & Offspring pooled		Logistic regression Sex-specific 1. birth cohort 2. multivariable adjusted for birth cohort, education, smoking, obesity (BMI ≥ 30), CVD risk factors, co-morbidity measured at exam 1
**Morbidity-free survival **(free of CVD, cancer and dementia) • At age 65 years (1. morbidityfree65X, 2. morbidityfree65MVX)	2	558 (558)	Cohort & Offspring pooled, exams closest to age 65 years		Logistic regression Sex-specific 1. birth cohort 2. multivariable adjusted for birth cohort, education, smoking, obesity, CVD risk factors measured at exam closest to age 65 years (within a 5 year horizon)

**Quantitative Traits: Linear regression**					

**Reproductive Aging** • Age at natural menopause (1. menoageX, 2. menoageMVX)	2	438 (378)	Cohort & Offspring pooled, women only		Linear regression 1. crude 2. multivariable adjusted for smoking, BMI, parity, generation (measured at exams prior to menopause)
**Cognitive function** • MMSE at age 65 years (1. MMSE65X, 2. MMSE65MVX) • MMSE at the specified Offspring exam (1. MMSE5X, 2. MMSE5MVX, 1. MMSE7X, 2. MMSE7MV, 1. MMSE5to7X, 2. MMSE5to7MVX)	2	593 (462)	Cohort & Offspring pooled, exams at age 65		Linear regression Sex-specific 1. birth cohort 2. multivariable adjusted for birth cohort, education, FSRP measured at exam closest to age 65 years (5 year horizon)
	6	1038 (913)	Exam 5 Exam 7 Exam 5 & 7 average score		Linear regression Sex-specific 1. birth cohort 2. multivariable adjusted for birth cohort, education, FSRP; covariates measured at the specified exam
**Physical Performance** • Hand grip (2. handgrip7x, 2. handgrips727x) • Walking speed (2. walkingspeed7x, 2. walkingspeed727x)	6	764	Exam 7 Exam 7 and Exam 27		Linear regression Sex-specific‡ 1. age 2. multivariable adjusted for age, height, weight at the specified exam
**Biologic Age by Osseographic Scoring System **(1. deltaOSSr, delta OSSrf, deltaOSSrm)	3	714	Offspring and Cohort pooled exam 6/7 and exam 22		Linear regression Sex- and cohort-specific ranked residuals§ 1. multivariable adjusted for age, height, BMI, menopause, estrogen use

Residuals from these models were used as traits to test for association with SNP genotypes.

* The number preceding the variable name refers to the covariate adjustment in the last column of the table. The website with all results is found at http://www.ncbi.nlm.nih.gov/projects/gap/cgi-bin/study.cgi?id=phs000007; ** MV = N for multivariable trait

‡ cohort- and sex-specific residuals for traits that included both cohort and offspring; §cohort-specific for traits limited to one sex

ALE = average life expectancy, BMI = body mass index, Co-morbidity = cardiovascular disease and cancer, CVD = cardiovascular disease, FSRP = Framingham stroke risk profile, MMSE = mini-mental state exam, Risk factors = hypertension, diabetes, elevated cholesterol

### SNP prioritization

We used several strategies to prioritize SNPs associated with longevity and aging traits. First, we used an untargeted approach whereby the top 50 SNP associations ranked according to the strength of the p-value for each trait were examined. Next, we explored the consistency of SNP associations across related sets of traits chosen a priori (trait set one: age at death and morbidity-free survival at age 65 years; trait set two: biologic age and walking speed). Trait set one was chosen based upon linkage data in humans demonstrating that both longevity and a healthy aging trait were linked to the same region on chromosome 4 raising the hypothesis that the two phenotypes may share common genetic pathways [11,12]. The traits in set two reflect aging with good physical functioning and thus we postulated that biologic age and walking speed may have genetic variants in common. We also investigated SNP associations in candidate genes and regions reported to be associated with longevity identified from established databases including NCBI [14] using the search term "longevity" and the Science of Aging Knowledge Environment genes/intervention database http://sageke.sciencemag.org/cgi/genesdb[30] choosing genes potentially related to lifespan in humans.

The SNPs were annotated using the UCSC genome browser tables using the May 2004 assembly http://genome.ucsc.edu/[31,32]. All genes within 60 kb of the top ranked SNPs were identified.

## Results

The longevity and aging traits available in the FHS 100K SNP resource are listed in Table 1. In this report, we consider only five of the traits listed in Table 1: multivariable-adjusted age at death, morbidity-free survival at age 65 years, age at natural menopause, walking speed, and biologic age by OSS. These traits include a pooled sample of Original Cohort and Offspring participants, with the exception of walking speed, which is reported in Offspring participants only. Details of the sample size and covariate adjustment for each trait are provided in Table 1.

For each of the five phenotypes, Table 2a and 2b provides the top five SNPs ranked in order by lowest p-value for the GEE and FBAT models (all associations can be viewed on the web http://www.ncbi.nlm.nih.gov/projects/gap/cgi-bin/study.cgi?id=phs000007). If multiple SNPs in linkage disequilibrium (LD r^2 ^> 0.80) were included in the top 5, additional SNPs were included until a set of 5 independent associations were listed. Eight SNPs on chromosome 1 were associated with age at death in the FBAT analysis; all with p-value < 10^-4 ^and two with p-value < 10^-5^. The 8 SNPs consisted of two sets of SNPs (rs10493513, rs10493514, rs6689491, rs6657082, rs1405051) and (rs10493515, rs10493518, rs10493517), clustered in two regions approximately 500 kb apart. There was exceptionally high LD across this 500 kb region: the minimum r^2 ^between pairs of the eight SNPs was 0.58. The nearest genes in this region existing in public databases were >500 kb from any of these SNPs [31,32].

**Table 2:** Aging and Longevity Phenotypes† for FHS 100K Project: Results of Association and Linkage Analyses

2a. GEE, Top 5 p-values by Phenotype*						
**Trait**	**SNP**	**Chromosome**	**Physical location**	**GEE p-value**	**FBAT p-value**	**Gene Region (within 60 kb)**

**Age at death**						
	rs1528753	11	90,523,987	**8.1 × 10^-8^**	0.024	
	rs2371208	7	81,982,510	**2.6 × 10^-6^**	0.031	
	rs10496799	2	139,261,401	**1.4 × 10^-5^**	0.735	NXPH2
	rs10489006	4	31,444,987	**3.6 × 10^-5^**	0.078	
	rs3757354	6	16,235,386	**6.4 × 10^-5^**	0.316	MYLIP
**Morbidity-free survival at age 65**						
	rs1412337	1	165,350,299	**1.8 × 10^-9^**	0.505	DPT
	rs32566	5	5,845,507	**1.9 × 10^-9^**	0.323	
	rs10484246	6	9,559,183	**8.4 × 10^-8^**	0.928	
	rs4831837	8	12,756,234	**4.7 × 10^-7^**	0.182	
	rs2639889	16	59,680,648	**9.4 × 10^-7^**	0.903	
**Age at natural menopause‡**						
	rs10496265	2	81,580,466	**1.1 × 10^-8^**	0.001	
	rs10496262*	2	81,662,782	**3.3 × 10^-7^**	0.005	
	rs958672	2	154,896,075	**1.9 × 10^-6^**	0.087	GALNT13
	rs291353	1	232,046,939	**5.5 × 10^-6^**	0.035	GNG4
	rs726336	5	163,911,906	**1.1 × 10^-5^**	0.125	
**Walking speed exam 7**						
	rs7137869	12	118,452,366	**6.3 × 10^-7^**	0.009	CCDC60
	rs7662116	4	154,375,569	**1.9 × 10^-5^**	0.016	
	rs7972859*	12	118,452,765	**2.5 × 10^-5^**	0.005	CCDC60
	rs9318312	13	74,489,506	**5.8 × 10^-5^**	0.266	
	rs1994854	4	78,124,824	**9.4 × 10^-5^**	0.280	
	rs7718104	5	122,183,258	**1.2 × 10^-4^**	0.011	SNX2
**Biologic age by osseographic scoring system**						
	rs1463605	12	30,005,150	**7.0 × 10^-8^**	5.3 × 10^-4^	
	rs7176093	15	84,170,434	**7.4 × 10^-6^**	0.005	KLHL25
	rs3772255	3	157,585,436	**8.2 × 10^-6^**	0.085	KCNAB1
	rs726846	5	136,099,953	**1.1 × 10^-5^**	0.003	
	rs646983	13	29,413,553	**1.2 × 10^-5^**	0.003	

2b. FBAT, Top 5 p-values by Phenotype*						

**Trait**	**SNP**	**Chromosome**	**Physical location**	**GEE p-value**	**FBAT p-value**	**Gene Region (within 60 kb)**

**Age at death**						
	rs10493513	1	73,091,610	0.640	**1.5 × 10^-6^**	
	rs10493514*	1	73,092,533	0.623	**2.8 × 10^-6^**	
	rs6689491*	1	73,064,050	0.205	**2.0 × 10^-5^**	
	rs10493515	1	73,527,652	0.225	**2.3 × 10^-5^**	
	rs10493518*	1	73,572,652	0.191	**3.6 × 10^-5^**	
	rs10493517*	1	73,570,372	0.215	**4.2 × 10^-5^**	
	rs6657082*	1	73,065,349	0.224	**5.5 × 10^-5^**	
	rs10498263	14	19,285,288	0.310	**8.3 × 10^-5^**	OR4Q3 OR4M1
	rs1915501	4	28,612,632	0.383	**1.1 × 10^-4^**	
	rs1405051*	1	73,060,505	0.176	**1.4 × 10^-4^**	
	rs6459623	6	18,634,791	0.604	**1.5 × 10^-4^**	IBRDC2
**Morbidity-free survival at age 65**						
	rs10509200	10	65,296,567	0.613	**7.0 × 10^-5^**	
	rs965036	6	20,099,022	0.550	**8.6 × 10^-5^**	
	rs720565	6	136,834,657	0.014	**9.8 × 10^-5^**	MAP7
	rs1192372	2	84,923,204	0.094	**9.9 × 10^-5^**	
	rs10505239	8	115,976,403	0.141	**1.3 × 10^-4^**	
**Age at natural menopause†**						
	rs959702	10	2,139,260	0.003	**1.4 × 10^-5^**	
	rs7165378	15	69,478,558	0.006	**6.4 × 10^-5^**	
	rs997161	10	130,876,127	0.074	**8.6 × 10^-5^**	
	rs165284	1	91,235,574	0.006	**8.6 × 10^-5^**	ZNF644
	rs2280585	3	64,882,324	0.884	**1.1 × 10^-4^**	
**Walking speed exam 7**						
	rs4471448	11	86,760,972	0.175	**3.8 × 10^-6^**	TMEM135
	rs336963	5	83,005,460	0.570	**2.6 × 10^-5^**	HAPLN1
	rs7862683	9	18,257,947	0.001	**4.9 × 10^-5^**	
	rs9317757	13	68,458,430	0.252	**7.8 × 10^-5^**	
	rs10501636*	11	86,789,967	0.355	**1.1 × 10^-4^**	
	rs2340392	3	80,440,990	0.002	**1.7 × 10^-4^**	
**Biologic age by osseographic scoring system**						
	rs1380703	2	57,852,938	0.008	**1.1 × 10^-5^**	
	rs324702	4	77,094,969	0.390	**3.3 × 10^-5^**	PPEF2
	rs324735	4	77,062,193	0.096	**7.6 × 10^-5^**	
	rs1106184	2	10,914,125	0.006	**8.8 × 10^-5^**	PDIA6
	rs604578	18	30,923,438	0.004	**9.5 × 10^-5^**	MAPRE2

**2c Linkage^¶ ^Peaks with LOD scores ≥ 2.0**						

**Trait**	**SNP closest to linkage peak**	**Chromosome**	**Physical location**	**1.5 – LOD support interval start**	**1.5 – LOD support interval end**	**LOD score**

**Age at natural menopause**						
	rs1371217	**4**	182,890,808	178,671,796	186,905,362	**2.08**
	rs10509024	**10**	56,567,832	36,084,470	70,573,011	**2.39**
	rs4793513	**17**	66,429,892	60,635,492	69,512,021	**2.48**
**Walking speed, exam 7**						
	rs2769261	**1**	113,278,949	107,080,550	144,332,709	**2.30**
	rs921055	**2**	233,522,842	229,328,008	242,141,304	**2.13**
	rs2602044	**3**	109,427,883	102,255,525	111,604,019	**3.38**
	rs8011773	**14**	96,927,485	96,342,135	100,389,787	**2.69**
	rs1362626	**16**	4,489,227	205,160	10,344,522	**2.05**
**Biologic age by osseographic scoring system**						
	rs353810	**9**	86,258,745	81,441,976	92,220,168	**3.26**
	rs1203981	**16**	205,160	205,160	7,431,239	**2.49**
	rs2248383	**21**	35,151,811	27,412,716	40,940,879	**2.22**

SNP criteria: Autosomal SNPs with genotypic call rate ≥ 80%, minor allele frequency ≥ 10%, Hardy-Weinberg test p > 0.001, and ≥10 informative families for FBAT

* For each phenotype SNPs are ranked by p-value. A SNP in LD (r^2 ^> 0.8) with a higher ranked SNP, is identified with an asterisk. All SNPs for a phenotype are listed until 5 independent SNPs are identified. Thus, for some phenotypes more than 5 SNPs are listed. For the age at death trait, the FBAT analysis identified two areas on chromosome 1 in LD, with r^2 ^= .5–.6 between the two regions and r^2 ^of nearly 1.0 within the region.

† Multivariable-adjusted trait results are presented

‡Trait had <500 participants in the sample.

¶Results limited to traits presented

There were several additional associations not listed in Table 2a and 2b that were of interest. For age at death in the GEE analysis, SNP associations ranked numbers 9 and 13 were rs10507486 (p-value 0.000128) and rs4943794 (p-value 0.000277), both are intronic *FOXO1A *SNPs. For age at natural menopause, top ranked SNP associations in the GEE model included number 11, rs6910534 (p = 0.00003) near *FOXO3a *and number 18, rs3751591 (p = 0.00006) in *CYP19A1*.

Table 2c presents the LOD scores ≥2.0 and the corresponding 1.5-LOD support interval from genome-wide linkage for the three quantitative aging traits. None of the regions overlapped with SNPs associated with these aging traits in the FBAT and GEE analyses. Of note for biologic age by OSS the linkage peak on chromosome 21 confirmed a prior Framingham Study report using a genome-wide scan with 401 microsatellite markers [10].

Table 3 provides all SNP associations with a GEE or FBAT p < 0.01 for both traits within the two pairs of related traits. For age at death and morbidity-free survival at age 65 years, FBAT models identified 7 SNPs and GEE models identified 9 SNPs associated with both traits including rs2374983 near *PON1 *(Tables 3a and 3b). For biologic age by OSS and walking speed, 13 SNPs in FBAT models and 6 SNPs in GEE models were associated with both traits (Tables 3c and 3d).

**Table 3:** All Significant SNP Associations (GEE or FBAT p-value < 0.01) for at least Two Traits

3a. FBAT: Age at Death and Morbidity-Free Survival at 65 years									
**Trait 1**	**Trait 2**	**SNP**	**Chr**	**Physical Position**	**Gene**	**Trait 1 GEE p-value**	**Trait 1 FBAT p-value**	**Trait 2 GEE p-value**	**Trait 2 FBAT p-value**

**Age at Death**	**Morbidity-free at 65 **	rs6682403	1	234,743,324		0.849	**0.004**	0.106	**0.004**
		rs10488907	4	113,669,709	ALPK1	0.452	**0.008**	0.336	**0.009**
		rs17190837	9	13,391,548		0.010	**0.009**	0.097	**0.004**
		rs4752977	11	47,257,005	**MADD**	0.736	**0.008**	0.002	**0.009**
		rs10506274	12	80,103,932		0.531	**0.001**	0.989	**0.001**
		rs2831154	21	28,059,331		0.323	**0.008**	0.358	**0.006**
		rs243725*	21	28,060,803		0.264	**0.007**	0.359	**0.008**

*r^2 ^> 0.80 with the preceding SNP									

**3b. GEE: Age at Death and Morbidity-Free Survival at 65 years**									

**Trait 1**	**Trait 2**	**SNP**	**Chr**	**Physical Position**	**Gene**	**Trait 1 GEE p-value**	**Trait 1 FBAT p-value**	**Trait 2 GEE p-value**	**Trait 2 FBAT p-value**

**Age at Death**	**Morbidity-free at 65 **	rs9308261	1	113,603,160	MAGI3	**0.009**	0.156	**0.002**	0.900
		rs10490518	2	31,223,004	GALNT14	**0.009**	0.373	**0.010**	0.948
		rs2374983	7	94,516,375	**PPP1R9A/PON1**	**0.006**	0.980	**0.007**	0.727
		rs655883	11	98,994,584	CNTN5	**0.006**	0.390	**0.001**	0.293
		rs1368850	11	130,433,518		**0.004**	0.387	**0.005**	0.292
		rs4943116	13	32,995,650	STARD13	**0.006**	0.205	**0.008**	0.049
		rs2254191	13	45,344,403		**0.007**	0.425	**0.004**	0.116
		rs1620210	13	45,759,488	C13orf18	**0.001**	0.161	**0.004**	0.068
		rs2823322	21	15,814,903		**0.0004**	0.045	**0.006**	0.029

**3c. FBAT: Biologic Age and Walking Speed**									

**Trait 1**	**Trait 2**	**SNP**	**Chr**	**Physical Position**	**Gene**	**Trait 1 GEE p-value**	**Trait 1 FBAT p-value**	**Trait 2 GEE p-value**	**Trait 2 FBAT p-value**

Biologic age	Walking speed	rs873348	4	178,246,509		0.135	**0.004**	0.114	**0.006**
Biologic age	Walking speed	rs10520361*	4	178,247,037		0.074	**0.006**	0.054	**0.005**
Biologic age	Walking speed	rs31564	5	135,258,152	IL9	0.015	**0.001**	**0.008**	**0.002**
Biologic age	Walking speed	rs1862345	5	148,018,498	HTR4	0.172	**0.0002**	0.399	**0.008**
Biologic age	Walking speed	rs7844834	8	11,323,556	C8orf12|C8orf13	0.017	**0.004**	0.011	**0.004**
Biologic age	Walking speed	rs952658	12	20,756,568	SLCO1C1	0.024	**0.008**	0.935	**0.006**
Biologic age	Walking speed	rs6487366	12	23,994,617	SOX5	0.017	**0.003**	0.959	**0.004**
Biologic age	Walking speed	rs7135493	12	28,134,847		0.020	**0.006**	0.033	**0.004**
Biologic age	Walking speed	rs10492036	12	124,728,934		0.308	**0.005**	0.169	**0.009**
Biologic age	Walking speed	rs1978945	13	105,641,257		0.093	**0.009**	0.031	**0.006**
Biologic age	Walking speed	rs2165723*	13	105,641,610		0.046	**0.010**	0.027	**0.003**
Biologic age	Walking speed	rs10492651*	13	105,641,634		0.083	**0.009**	0.023	**0.001**
Biologic age	Walking speed	rs9301112*	13	105,642,018		0.097	**0.004**	0.053	**0.003**

* r^2 ^> 0.8 with the preceding SNP (calculated if the distance is <250,000 base pairs)									

**3d. GEE: Biologic Age and Walking Speed**									

**Trait 1**	**Trait 2**	**SNP**	**Chr**	**Physical Position**	**Gene**	**Trait 1 GEE p-value**	**Trait 1 FBAT p-value**	**Trait 2 GEE p-value**	**Trait 2 FBAT p-value**

Biologic Age	Walking speed	rs1474827	6	134,886,011		**0.007**	0.746	**0.000**	0.233
Biologic Age	Walking speed	rs10231641	7	119,166,342		**0.004**	0.077	**0.009**	0.278
Biologic Age	Walking speed	rs310575	8	51,603,257	SNTG1	**0.008**	0.209	**0.003**	**0.007**
Biologic Age	Walking speed	rs10520603	15	84,170,955		**0.009**	**0.008**	**0.003**	0.050
Biologic Age	Walking speed	rs7166323*	15	84,171,745		**0.009**	0.012	**0.003**	0.065
Biologic Age	Walking speed	rs2215921	16	9,604,834		**0.007**	0.049	**0.001**	0.316

* r^2 ^> 0.8 with the preceding SNP (calculated if the distance is <250,000 base pairs)									


We identified from the literature 79 potential candidate genes and regions associated with longevity (see Additional file 1 for listing). Of these, 12 genes had no SNPs and 67 genes had 1 to 45 SNPs within 60 kb of the gene on the 100K Affymetrix GeneChip. There were 2036 SNPs in the LGV1 region on chromosome 4 previously linked to exceptional longevity [11]. Table 4 shows the candidate genes with SNPs associated with an FBAT or GEE p-value < 0.01 for age at death including: *FOXO1a, GAPDH, KL, LEPR, PON1, PSEN1, SOD2, and WRN *and for morbidity-free survival at 65 years including:*GHR, LEPR, MORF4L1, PON1, PTH, and WRN*. Biologic age by OSS shared 2 SNPs in common with age at death: rs4943794 intronic to *FOXO1a *and rs911847 near *SOD2*.

**Table 4:** All Significant SNP Associations with Selected Longevity Candidate Genes* (FBAT or GEE p-value < 0.01)

Trait	Gene	SNP	Chr	Physical Position	FBAT p-value	GEE p-value	SNP function	SNP position relative to gene (up to 60 kb)
**Age at death**	**FOXO1a**	**rs4943794**	13	40,071,408	0.068	**0.00028 **	Intron	in
		rs10507486	13	40,084,501	0.043	**0.00013 **	Intron	in
	**GAPDH†**	rs4764600	12	6,472,241	0.833	**0.005**	Locus/intron	near
	**KL**	rs683907	13	32,522,175	**0.009**	0.507	Intron	in
		rs687045	13	32,522,889	**0.007**	0.712	Intron	in
	**LEPR**	rs1475398	1	65,695,278	0.069	**0.005**	Untranslated	in
		rs1343981	1	65,757,349	0.031	**0.006**	Intron	in
		rs10493379	1	65,757,948	0.015	**0.004**	Intron	in
		rs2154380	1	65,769,462	**0.004**	**0.003**	Intron	in
		rs6669117	1	65,773,093	0.050	**0.007**	Intron	in
	**PON1**	**rs2374983**	7	94,516,375	0.980	**0.006**	Intron	near
	**PSEN1**	rs362356	14	72,708,382	**0.005**	0.130	Intron	in
	**SOD2**	**rs911847**	6	160,039,379	0.358	**0.005**	Unknown	near
	**WRN‡**	rs2543600	8	30,969,282	0.182	**4.2 × 10^-6^**	Unknown	near
**Morbidity-free survival at age 65**	**GHR**	rs719756	5	42,761,386	**0.003**	0.676	Unknown	near
	**LEPR**	rs1171278	1	65,700,167	0.042	**0.003**	Untranslated	in
		rs3790426	1	65,755,040	0.460	**0.002**	Intron	in
	**MORF4L1**	rs1383636	15	76,893,275	0.458	**0.007**	Unknown	near
	**PON1**	**rs2374983**	7	94,516,375	0.727	**0.007**	Intron	near
		rs854523	7	94,542,884	0.850	**0.007**	Intron	in
	**PTH**	rs10500784	11	13,530,401	**0.010**	0.990	Unknown	near
	**WRN‡**	rs2725369	8	30,970,566	0.113	**0.003**	Unknown	near
**Biologic Age by OSS**	**FOXO1a**	rs1923249	13	40,041,881	**0.006**	**0.004**	Intron	in
		**rs4943794**	13	40,071,408	**0.009**	0.016	Intron	in
	**HSPA9**	rs256014	5	137,930,983	0.101	**0.005**	Intron	in
	**LASS6**	rs1002666	2	169,303,525	**0.001**	**0.008**	Intron	in
	**SOD2**	**rs911847**	6	160,039,379	0.024	**0.009**	Unknown	near
	**TLR4**	rs1927914	9	117,544,279	**0.007**	0.401	Locus	near
**Walking speed**	**ESR1**	rs9322361	6	152,551,257	0.124	**0.0089 **	Intron	in
	**LASS6**	rs6433083	2	169,324,821	0.232	**0.006**	Intron	in
	**NR3C1**	rs2918418	5	142,703,566	**0.005**	0.081	Intron	in
		rs10515522	5	142,738,587	**0.004**	0.084	Intron	in
	**SOD1**	rs2833485	21	32,000,796	**0.008**	0.507	Locus/intron	in
	**TERF2**	rs728546	16	68,013,029	**0.0045**	0.533	Unknown	near
	**FASLG**	rs6700734	1	169,362,468	**0.003**	0.029	Intron	in

*79 genes identified from NCBI, SAGE ke, and GenAge databases; 12 genes with no SNPs on 100K chip; 67 genes with 1–45 SNPs on 100K chip; LGV1 2036 SNPs on 100K chip, results for this region available on the web

†The most strongly associated SNP near GAPDH is actually closer to MRPL51

‡The most strongly associated SNP near WRN is actually closer to PURG

## Discussion

To our knowledge, this is the first dense GWAS of longevity and aging traits in a community-based sample of adults from two generations of the same families. Over 1300 men and women have detailed longevity and aging-related phenotypes and 100K SNP genotyping results available on the web. This resource has the potential to detect novel susceptibility genes for human longevity and aging and to examine the relevance of promising candidate gene associations reported in animal models to human aging. We describe several strategies to prioritize SNP associations in this unique resource to enhance the discovery of various genes and pathways that contribute to the control of human longevity. Furthermore, FHS investigators are part of the NIA sponsored Longevity Consortium http://www.longevityconsortium.org which offers the opportunity of collaboration with other investigators to replicate important findings in additional cohorts.

In our untargeted approach of ranking SNP associations by the strength of the p-value, 2 intronic *FOXO1a *SNPs were associated with age at death. One of these SNPs (rs4943794) also was associated with biologic age by OSS in our a priori evaluation of select candidate genes. FOXO (forkhead box group O) transcription factors are targets of insulin-like signaling and are involved in a diverse set of physiological functions including DNA repair and resistance to oxidative stress [33,34]. Further, FOXO plays a role in lifespan extension in *C. elegans *and *Drosophila *[35]. Studies of this gene in humans are limited; two case-control studies have not identified an association between *FOXO1a *and longevity [36,37]. However, the prospective population-based Leiden 85-plus Study found that *FOXO1a *was associated with increased mortality attributable to diabetes related deaths in participants aged 85 years and older [38]. The Leiden 85-plus Study also reported that genetic variation causing a reduction in insulin/IGF-1 signaling resulted in improved old age survival among women [20]. However, that report examined other genes in the insulin/insulin-like signaling pathway and did not specifically examine *FOXO1a*. Finally, the untargeted approach to SNP selection also identified a SNP near *FOXO3a *associated with age at natural menopause. This gene has been implicated in oocyte death, depletion of functioning ovarian follicles, and infertility in mice [39,40] and thus represents a plausible candidate gene for menopause. Most positive common gene variant-disease association studies have failed replication [41] including reports on exceptional longevity. Haplotype-based fine mapping of the region on chromosome 4 linked to human longevity initially suggested the *MTP *gene, a gene important in lipoprotein synthesis, was associated with longevity [21]. However, this association failed replication in a French cohort of long-lived individuals and subsequent case-control studies of nonagenarians [22,42]. Beekman, *et al *[43] found neither linkage to chromosome 4 nor association with the MTP gene and longevity among nonagenarians in the Leiden Longevity Study. Meta-analyses implicated admixture of the control sample in the original report as an explanation for the presumed false-positive association. Thus, our findings are hypothesis generating and their importance can not be determined without evidence of consistent replication in other populations.

We examined pleiotropic effects by identifying SNP associations across two pairs of related traits. One SNP near *PON1 *emerged as associated with both age at death and morbidity-free survival. Surprisingly, there were relatively few SNPs associated with both traits; prior work had suggested that longevity per se and healthy aging may share common genetic pathways [11,12]. However, morbidity-free survival was measured at age 65 years, it is possible that as our participants age morbidity-free survival defined at age 75 or 85 years will share additional SNP associations with our longevity trait, age at death. A SNP near *SOX5*, a gene potentially related to musculoskeletal function was associated with both biologic age by OSS and walking speed.

Our strategy of selecting SNPs in candidate genes and regions previously reported to be associated with longevity yielded interesting findings. For age at death, we identified SNPs in or near several genes including *KL, LEPR, PON1, SOD2*, and *WRN*. Defects in the *WRN *gene are the cause of Werner Syndrome, an autosomal recessive disorder characterized by premature aging. A longitudinal study of ageing Danish twins recently reported a possible association between a successful aging trait and 3 SNPs in the *WRN *gene [44]. We were unable to determine if our SNP (rs2725369) was in LD with the SNPs in the prior report because the SNPs were not included in HapMap. Mutations in the *KL *(Klotho) gene in the mouse lead to a syndrome resembling human aging [45-47]. There has been one report linking a functional variant of the *KL *gene to human longevity [15]. Thus, results from this GWAS may direct resources to the most relevant candidate genes and pathways for further investigation in humans.

Several important limitations merit comment. First, we acknowledge that there may be a survival bias as participants in this sample had to survive to provide DNA (first systematic DNA collection began 1995) and hence are likely healthier than the full FHS sample. To ameliorate this issue, we adjusted for covariates using the full Framingham sample, and used the residual traits for the subset of individuals genotyped using the 100K Affymetrix GeneChip to test for association with the SNPs using linear regression models. Residual traits from Cox and logistic models typically are not ideally distributed for linear regression models, but our adjustment method using the full sample precludes the testing of SNP associations with age at death and morbidity-free survival using Cox and logistic models. Second, the 100K Affymetrix GeneChip provides limited coverage of the genome; many of our a priori candidate genes did not have any SNP coverage on the chip. For example, several genes that have been studied in model organisms or even in humans such as *ACE, Lamin A, SIRT2 *and *SIRT3*, had no SNPs within 60 kb of the gene on the 100K Affymetrix GeneChip. However, genotyping is near-complete for the NHLBI funded 550 K genome-wide scan on all FHS participants. This will enable deeper exploration of our initial 100K SNP associations in a larger sample with denser coverage of the genome. Third, in this analysis we did not examine epistasis or gene-environment interactions which may modify the associations in this study. Importantly, this study is hypothesis generating. Our findings need to be replicated in other samples.

## Conclusion

In summary, the untargeted genome-wide approach to detect genetic associations with longevity and aging traits provides an opportunity to identify novel biologic pathways related to lifespan control. GWAS also have the potential to direct investigators of human aging to the most promising candidate gene associations and biologic pathways reported to regulate lifespan in animal models. Enhancing our understanding of the mechanisms responsible for aging may in turn identify directions for health promotion and disease prevention efforts in middle-aged and older adults so that older persons can enjoy more time in good health. These data generate hypotheses regarding novel biologic pathways contributing to longevity and healthy aging and serve as a resource for replication of findings from other population-based samples.

## Abbreviations

CVD = cardiovascular disease; FBAT = family-based association test; FHS = Framingham Heart Study; GEE = generalized estimating equations; GWAS = genome-wide association study; LD = linkage disequilibrium; LOD = logarithm of the odds; NCBI = National Center Biotechnology Information; OSS = osseographic scoring system; SNP = single nucleotide polymorphism; TIA = transient ischemic attack.

## Competing interests

The authors declare that they have no competing interests.

## Authors' contributions

All authors have made substantial contributions to conception and design or acquisition of phenotypic data. JMM, KL, EJB, CG, DK, DPK, JMM, MJP, RBD contributed to the analysis and interpretation of data. JMM, KL, EJB, DK, DPK, SS have been involved in drafting the manuscript or revising it critically for important intellectual content. All authors read and approved the final manuscript.

## Supplementary Material

Additional file 1 Candidate Gene List for FHS 100K Longevity and Aging Traits Click here for file
