# Supplementary material for: Genetic correlates of longevity and selected age-related phenotypes: a genome-wide association study in the Framingham Study
Source: BMC Med Genet. 2007 Sep 19;8(Suppl 1):S13. doi: 10.1186/1471-2350-8-S1-S13 (PMC1995604; doi:10.1186/1471-2350-8-S1-S13)
Supplement: Additional file 1 — Candidate Gene List for FHS 100K Longevity and Aging Traits [file 1471-2350-8-S1-S13-S1.doc]

**Additional file 1: Candidate Gene List for FHS 100K Longevity and Aging Traits**

| **Gene** | **Gene Name** | **Number of SNPs within X base pairs of the gene** | | | | |
| --- | --- | --- | --- | --- | --- | --- |
|  |  | **0** | **2500** | **5000** | **50000** | **100000** |
| **ADIPOQ** | **Adiponectin, Adipocyte, C1Q, & Collagen Domain Containing** | 0 | 0 | 0 | 0 | 8 |
| **ACE** | **Angiotensin I-converting enzyme** | 0 | 0 | 0 | 0 | 0 |
| **AKAP10** | **A-Kinase Anchor Protein 10** | 1 | 1 | 1 | 1 | 1 |
| **APBB1** | **amyloid beta (A4) precursor protein-binding, family B, member 1** | 1 | 1 | 1 | 4 | 15 |
| **APOC3** | **ApolipoproteinC-III** | 0 | 0 | 0 | 1 | 7 |
| **APOE** | **Apolipoprotein E*** | 0 | 0 | 0 | 1 | 1 |
| **APP** | **Amyloid Beta A4 Precursor Protein** | 15 | 16 | 17 | 21 | 28 |
| **ATM** | **Ataxia telangiectasia mutated** | 2 | 2 | 2 | 4 | 4 |
| **BAX** | **BCL2-Associated X protein** | 0 | 0 | 0 | 0 | 0 |
| **BCL2L1** | **BCL2-like 1** | 0 | 0 | 0 | 0 | 0 |
| **BLM** | **Bloom syndrome** | 0 | 0 | 0 | 0 | 0 |
| **CETP** | **Cholesterol Ester Transfer Protein** | 0 | 0 | 0 | 1 | 4 |
| **CLU** | **Clusterin** | 1 | 1 | 1 | 6 | 7 |
| **COQ7** | **COQ7, S. CEREVISIAE, homolog of** | 0 | 0 | 0 | 0 | 1 |
| **DAF** | **Decay accelerating factor for complement (CD55)** | 0 | 0 | 0 | 7 | 13 |
| **EIF4G2** | **Eukaryotic Translation Initiation factor 4-Gamma, 2** | 0 | 2 | 2 | 6 | 8 |
| **ERCC8** | **excision repair cross-complementing rodent repair deficiency, complementation group 8** | 3 | 3 | 3 | 4 | 13 |
| **ESR1** | **Estrogen receptor 1** | 15 | 15 | 15 | 24 | 27 |
| **FHL2** | **Four-and-a-half LIM domains 2** | 4 | 4 | 4 | 5 | 10 |
| **FOXO1A** | **Forkhead Box O1A** | 5 | 5 | 5 | 5 | 8 |
| **FOXO3A** | **Forkhead Box O3A** | 5 | 5 | 5 | 9 | 15 |
| **GAPD** | **Glyceraldehyde-3-Phosphate Dehydrogenase** | 9 | 10 | 10 | 15 | 25 |
| **GH1** | **Growth hormone 1** | 0 | 0 | 0 | 1 | 1 |
| **GHR** | **Growth hormone 1 receptor** | 3 | 3 | 3 | 7 | 13 |
| **HSPA9** | **Heat shock 70kDa protein 9B (mortalin-2, Mot-2)** | 1 | 1 | 1 | 3 | 5 |
| **IGF1** | **Insulin-like growth factor 1** | 1 | 1 | 1 | 6 | 14 |
| **IGF1R** | **Insulin-like growth factor 1 receptor** | 7 | 7 | 7 | 7 | 7 |
| **IGFBP2** | **Insulin-like growth factor binding protein 2** | 0 | 0 | 0 | 0 | 1 |
| **IL10** | **Interleukin 10** | 1 | 1 | 1 | 4 | 8 |
| **IL6** | **Interleukin 6, interferon, beta 2** | 2 | 2 | 2 | 6 | 10 |
| **KL** | **Klotho** | 6 | 6 | 6 | 12 | 18 |
| **LASS1** | **LAG1 longevity assurance homolog 1 (S. cerevisiae)** | 0 | 0 | 0 | 0 | 0 |
| **LASS2** | **LAG1 longevity assurance homolog 2 (S. cerevisiae)** | 0 | 1 | 1 | 1 | 2 |
| **LASS3** | **LAG1 longevity assurance homolog 3 (S. cerevisiae)** | 5 | 5 | 5 | 8 | 9 |
| **LASS4** | **LAG1 longevity assurance homolog 4 (S. cerevisiae)** | 1 | 1 | 1 | 1 | 1 |
| **LASS5** | **LAG1 longevity assurance homolog 5 (S. cerevisiae)** | 0 | 0 | 0 | 1 | 1 |
| **LASS6** | **LAG1 longevity assurance homolog 6 (S. cerevisiae)** | 21 | 21 | 21 | 28 | 33 |
| **LEP** | **Leptin** | 0 | 0 | 1 | 2 | 2 |
| **LEPR** | **Leptin receptor** | 11 | 12 | 12 | 18 | 25 |
| **LGV1** | **Longevity 1** | 2022 | 2022 | 2022 | 2027 | 2036 |
| **LMNA** | **Lamin A** | 0 | 0 | 0 | 0 | 2 |
| **MLLT7** | **Myeloid/lymphoid or mixed-lineage leukemia (trithorax homolog, Drosophila);** | 0 | 0 | 0 | 0 | 0 |
| **MORF4** | **Mortality factor 4** | 0 | 0 | 0 | 2 | 5 |
| **MT2A** | **Metallothionein 2A** | 0 | 0 | 2 | 7 | 7 |
| **MTHFR** | **Methylenetetrahydrofolate reductase** | 1 | 1 | 1 | 1 | 1 |
| **MTP** | **Microsomal triglyceride transfer protein** | 2 | 2 | 2 | 13 | 21 |
| **NR3C1** | **nuclear receptor subfamily 3, group C, member 1** | 9 | 9 | 9 | 12 | 16 |
| **PARK2** | **Parkin** | 42 | 42 | 43 | 43 | 45 |
| **PON1** | **Paraoxonase 1** | 2 | 4 | 4 | 8 | 15 |
| **PPARA** | **Peroxisome proliferative activated receptor, alpha** | 0 | 0 | 0 | 0 | 0 |
| **PPARG** | **Peroxisome proliferative activated receptor, gamma** | 19 | 20 | 20 | 20 | 26 |
| **PRKCA** | **Protein kinase C, alpha** | 22 | 22 | 22 | 24 | 27 |
| **PSEN1** | **Presenilin 1 (Alzheimer disease 3)** | 5 | 5 | 5 | 5 | 10 |
| **PSEN2** | **Presenilin 2** | 3 | 3 | 3 | 4 | 11 |
| **PTH** | **Parathyroid hormone** | 1 | 1 | 3 | 7 | 16 |
| **S100B** | **S100 calcium binding protein, beta (neural)** | 2 | 2 | 2 | 2 | 5 |
| **SHC1** | **SHC transforming protein 1** | 0 | 0 | 0 | 0 | 1 |
| **SIRT2** | **Sirtuin 2 (S. cerevisiae)** | 0 | 0 | 0 | 0 | 0 |
| **SIRT3** | **Sirtuin 3** | 0 | 0 | 0 | 0 | 0 |
| **SLC6A4** | **Solute carrier family 6, member 4** | 0 | 0 | 0 | 0 | 0 |
| **SNCA** | **Synuclein, alpha** | 11 | 12 | 12 | 25 | 30 |
| **SOD1** | **Superoxide dismutase 1, soluble** | 2 | 2 | 2 | 7 | 12 |
| **SOD2** | **Superoxide dismutase 2, mitochondrial** | 0 | 0 | 0 | 1 | 3 |
| **TERF2** | **Telomeric repeat binding factor 2** | 0 | 0 | 0 | 2 | 2 |
| **TERT** | **Telomerase reverse transcriptase** | 0 | 0 | 0 | 0 | 0 |
| **TGFB1** | **Transforming growth factor, beta 1** | 0 | 0 | 0 | 0 | 1 |
| **TH** | **Tyrosine hydroxylase** | 0 | 0 | 0 | 0 | 0 |
| **TLR4** | **Toll-like receptor 4** | 0 | 1 | 3 | 6 | 15 |
| **TMEM57** | **Transmembrane protein 57** | 0 | 0 | 0 | 0 | 1 |
| **TNFRSF6** | **Fas (TNF receptor superfamily, member 6)** | 9 | 10 | 10 | 15 | 25 |
| **FASLG** | **Fas ligand (TNF superfamily, member 6)** | 1 | 1 | 1 | 4 | 4 |
| **TP53** | **Tumor protein p53 (Li-Fraumeni syndrome)** | 0 | 0 | 0 | 1 | 1 |
| **TRAM2** | **Translocation associated membrane protein 2** | 10 | 10 | 10 | 13 | 22 |
| **UCHL1** | **Ubiquitin Carboxyl-Terminal Esterase L1** | 5 | 5 | 5 | 6 | 10 |
| **VDR** | **Vitamin D (1,25- dihydroxyvitamin D3) receptor** | 5 | 5 | 5 | 5 | 5 |
| **WRN** | **Werner Syndrome** | 8 | 8 | 8 | 15 | 17 |
| **YTHDF2** | **YTH domain family, member 2** | 0 | 0 | 0 | 0 | 2 |

*Neither SNP within 100kb of APOE is in LD with the SNP in HapMap that partially defines the ε2/ε3/ε4 polymorphism.
